# The development of functional glutamatergic and GABAergic synaptic connections between vestibulo-ocular projection neurons and oculomotor motoneurons in the chicken embryo

**DOI:** 10.3389/fneur.2025.1568926

**Published:** 2025-07-21

**Authors:** Hiraku Mochida, Joel C. Glover

**Affiliations:** Section of Physiology, Department of Molecular Medicine, University of Oslo, Oslo, Norway

**Keywords:** vestibulo-ocular reflex (VOR), calcium green dextran amine, abducens interneurons, superior rectus, medial rectus, inferior oblique, inferior rectus

## Abstract

**Introduction and methods:**

We assessed the functional development of synapses from defined vestibulo-ocular projection neurons to motoneurons (MNs) in the oculomotor nuclear complex in the chicken embryo using optical recording of postsynaptic responses with the calcium-sensitive probe Calcium Green Dextran Amine (CGDA). The vestibulo-ocular projection neuron groups were defined according to the hodological nomenclature established by Díaz et al. (1) and encompassed the ipsilateral rostral, ipsilateral caudal and contralateral caudal vestibulo-ocular (iR-VO, iC-VO and cC-VO) groups. These groups provide differential input to the inferior rectus (IR), superior oblique (SO), superior rectus (SR) and inferior oblique IO) MN pools. The cC-VO group includes abducens interneurons (abd INs) which innervate the medial rectus (MR) MN pool. Since the SO MN pool, which projects out the trochlear nerve, was not labeled, recordings were limited to the IR, SR, IO and MR MN pools.

**Results:**

Single pulse stimulation of all the presynaptic axons collectively in wholemount preparations of the brain stem elicited robust calcium responses in all four of the CGDA-labeled postsynaptic MN pools. Responses were recorded from the dorsal surface in different regions of interest (ROIs) deployed along the mediolateral axis, which allowed us to distinguish responses in IR, MR+IO and SR MNs. Responses were first detected in a fraction of preparations at d7 of development, and from d8 in all preparations. Response magnitudes increased through d9, and then diminished through d11. Pharmacological experiments showed that responses included both glutamatergic (AMPA receptor and NMDA receptor-mediated) and GABAergic (GABA_A_ receptor-mediated) components. All three components were present when the first responses appeared, but their proportional contribution changed during development. Covert NMDA-sensitive responses could be revealed by superfusing unresponsive d7 preparations with Mg^2+^-free Ringer, indicating that the very first responses to develop were mediated by NMDA receptors. GABAergic responses involved the activation of voltage-gated calcium channels and were therefore likely to be depolarizing, but nevertheless had an inhibitory effect on glutamatergic responses. Paired pulse and train stimulation at d9-d11 demonstrated substantial facilitation of the aggregate response.

**Discussion:**

These data provide new information about the functional development of vestibulo-ocular reflex (VOR) circuitry and set the stage for testing the role of specific receptors and downstream signaling in establishing the specific synaptic connections that characterize the VOR.

## Introduction

**Figure 1:**
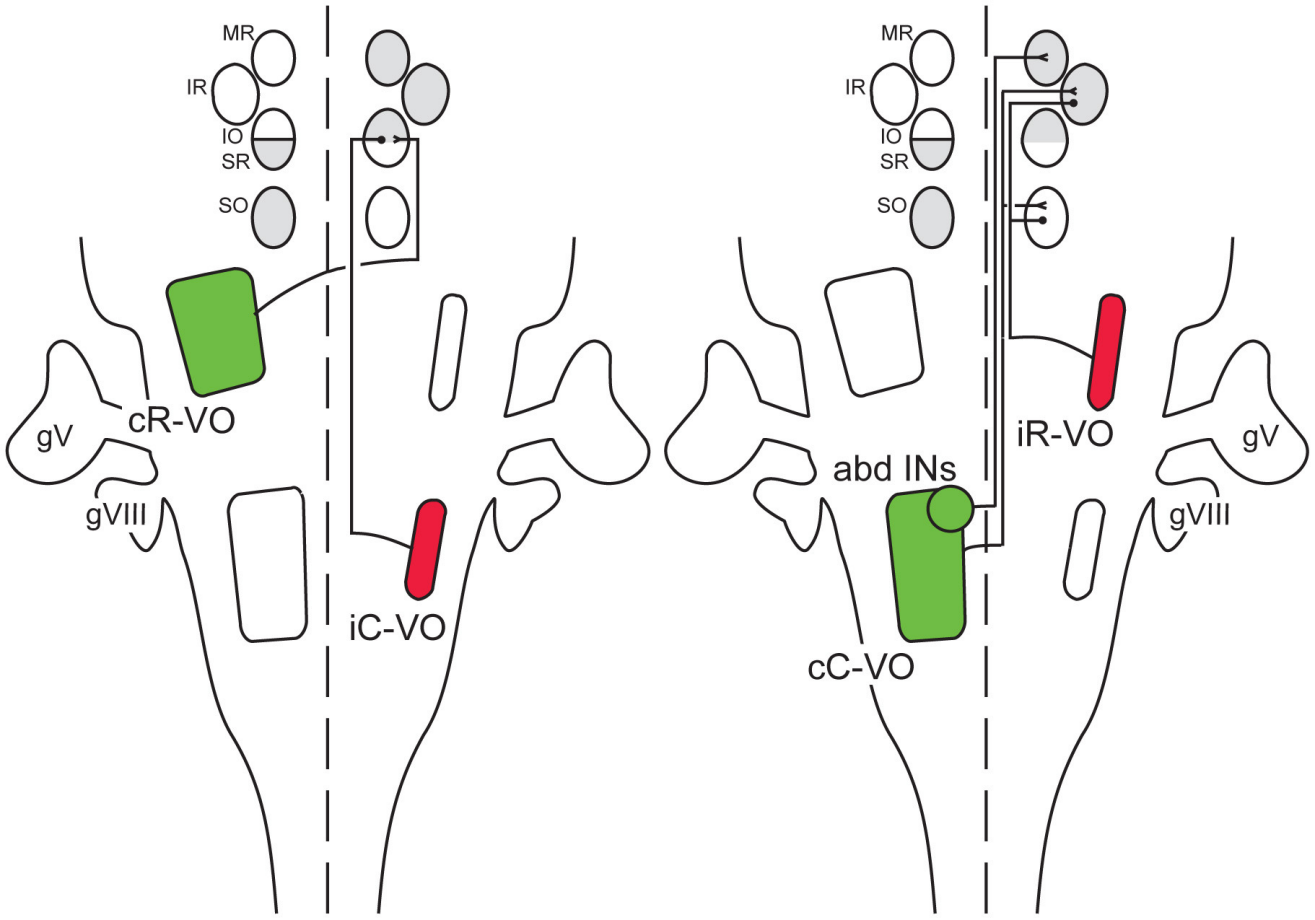
Schematic overview of hodologically defined vestibulo-ocular projection neuron groups that innervate extraocular motoneurons (MNs) in the oculomotor and trochlear nuclear complex. The approximate domain of each vestibulo-ocular projection neuron group is outlined on a frontal view of the brain stem, along with the corresponding axon trajectory and termination pattern among the extraocular MN pools (MR, medial rectus; IR, inferior rectus; IO, inferior oblique; SR, superior rectus; SO, superio2r oblique). The MN pools are color coded according to the laterality of their peripheral termination on the corresponding muscle (white = ipsilateral, gray = contralateral). Vestibulo-ocular projection neuron groups are named according to the hodological nomenclature of Díaz et al. (3): cR-VO, contralateral rostral vestibulo-ocular; iC-VO, ipsilateral caudal vestibulo-ocular, iR-VO, ipsilateral rostral vestibulo-ocular; cC-VO, contralateral caudal vestibulo-ocular; abd INs, abducens interneurons). They are further color-coded according to known or presumed functional effect (green = excitatory, red = inhibitory). gV, trigeminal ganglion; gVIII, vestibular ganglion.

Vestibulo-ocular reflex pathways mediate stereotyped connections from vestibular projection neurons to motoneurons (MNs) innervating extraocular muscles. These connections transfer sensory signals from vestibular end organs to pairs of extraocular MN pools in a system that ensures conjugated eye movements, in which the two eyes move in the same direction in space. For example, excitatory inputs to MNs innervating the lateral rectus (LR) muscle in one eye also activate interneurons in the abducens nucleus that in turn activate MNs innervating the medial rectus (MR) in the other eye, ensuring conjugate movements in the horizontal plane. Starting in the 1990s, it became clear that vestibular projection neurons that drive eye movements in the vertical and frontal (relative to the eyeball) planes are organized into spatially distinct clusters (hereafter referred to as groups), each of which innervates a specific pair of MN pools within the framework of conjugate eye movements (1, 2) (Figure 1). Thus, using the nomenclature introduced by Díaz et al. (3), neurons in the cR-VO (contralateral Rostral Vestibulo-Ocular) group excite MNs innervating the inferior oblique (IO) muscle in one eye and the superior rectus (SR) muscle in the other eye, and neurons in the cC-VO (contralateral Caudal Vestibulo-Ocular) group (minus the abducens interneurons, abINs, which occupy a specific domain within the cC-VO group) excite MNs innervating the superior oblique (SO) muscle in one eye and the inferior rectus (IR) muscle in the other eye. Moreover, additional groups of vestibulo-ocular neurons provide inhibitory input in the same paired fashion. Neurons in the iC-VO group inhibit MNs innervating the IO and SR muscles in opposite eyes, and neurons in the iR-VO group inhibit MNs innervating the SO and IR muscles in opposite eyes. This combination of excitatory and inhibitory inputs creates a “push-pull” organization in which the cR-VO and iC-VO neurons act antagonistically on the conjugate IO and SR MNs, and the cC-VO and iR-VO neurons act antagonistically on the conjugate SO and IR MNs.

Although this organization has been characterized most comprehensively in the chicken embryo (1), the same hodologically defined groups of VO neurons have been described in the mouse (4) and frog (5, 6), and all but the iC-VO group in the goldfish (7), suggesting a system that has been largely conserved in evolution from bony fish to mammals. An additional excitatory projection, the excitatory ascending tract of Deiters (ATD) from the lateral vestibular nucleus to MR MNs, has been demonstrated in the cat (8–10). The hodological organization described in the chicken embryo does not include the ATD projection, but nevertheless provides a complete functional push-pull organization that could engender conjugate eye movements in any plane through combinatorial activation of the characterized vestibulo-ocular neuron groups by sensory inputs (reviewed in 3).

The hodological patterning of the vestibulo-ocular projections and the developmental timing of synaptic contacts in the VOR pathway have been documented previously (1), but little is known about the functional development of the synaptic connections. Here, we assess the functional development of synapses between the vestibulo-ocular neuron groups and their target MNs in the oculomotor nucleus. To get around the technical difficulty of making electrophysiological recordings at early developmental stages, we have used an optical recording approach, relying on the labeling of specific MN pools with a calcium-sensitive tracer and the generation of postsynaptic intracellular calcium transients by synaptic activation. Intracellular calcium transients can arise either through the transmitter-mediated opening of calcium-permeable ionotropic receptors (such as the NMDA receptor), through the indirect opening of voltage-sensitive calcium channels in the cell membrane (which can be triggered by both glutamatergic, GABAergic and glycinergic synapses in the embryo due to the depolarizing nature of inhibitory inputs at embryonic stages; Gonzalez-Islas et al. (39)), or through the activation of calcium release from intracellular stores triggered by second-messenger systems. The optical approach has allowed us to record selectively from different oculomotor MN pools, and, in combination with pharmacological manipulations, to discriminate inputs mediated by GABA and glutamate and, in the case of glutamate, through different receptors (NMDARs vs. AMPARs). Although the approach does not allow us to characterize such features as postsynaptic potential amplitudes, waveforms or reversal potentials, it does allow us to create a timeline of functional excitatory and inhibitory synaptogenesis in the oculomotor MN pools, and to compare excitatory and inhibitory inputs semi-quantitatively in terms of their relative contributions to the resultant intracellular calcium transients. Thus, the results presented here provide new information about the timing of functional synaptogenesis between vestibulo-ocular neurons and target MNs and provide a platform for more detailed studies of synaptogenesis in the developing VOR pathway.

## Materials and methods

### Animals, *ex vivo* preparations and labeling with conjugated dextrans

**Figure 2:**
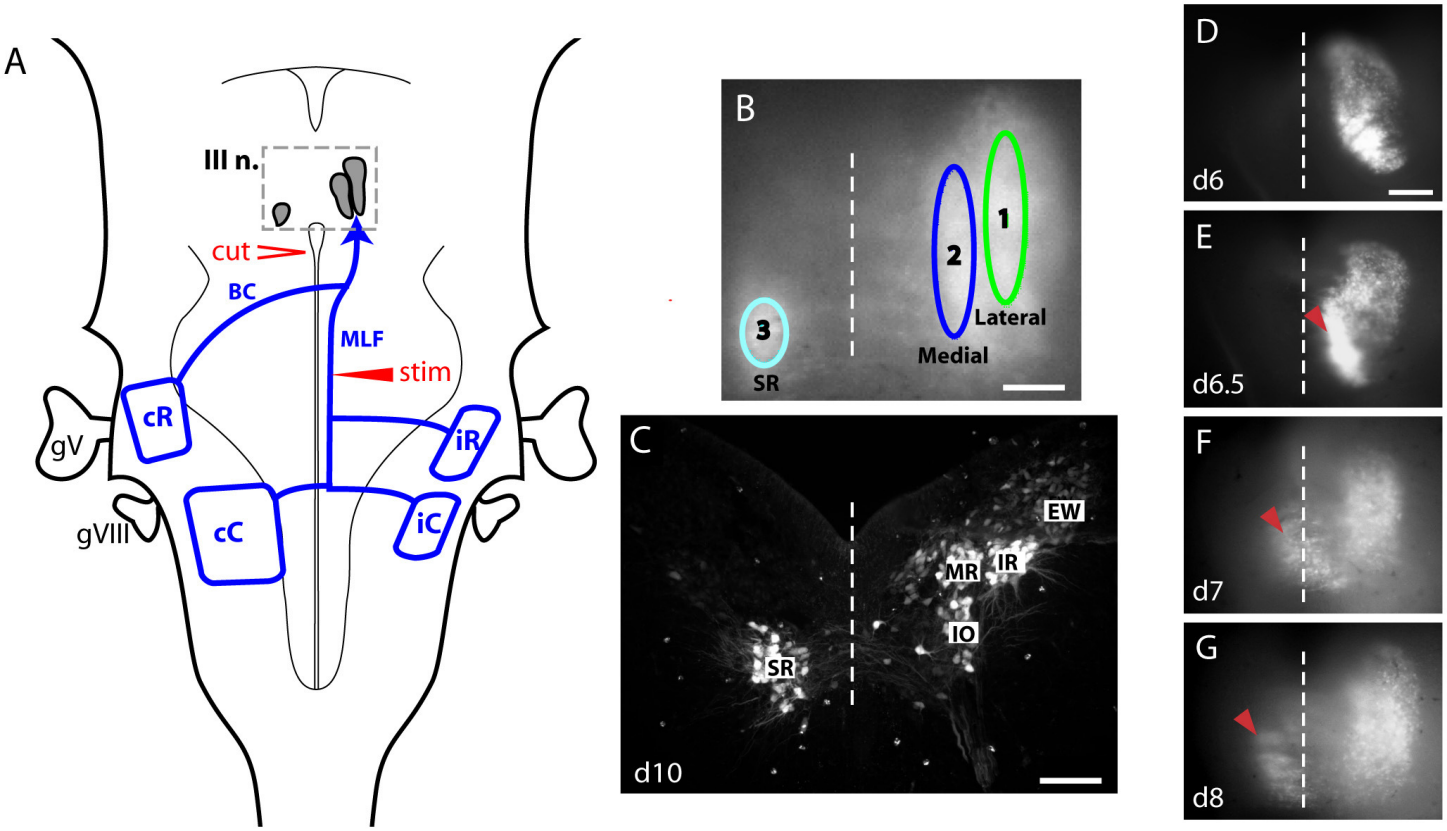
Overview of the experimental setup. **(A)** Schematic drawing of the dorsal surface of the hindbrain showing the relative locations of the CGDA-labeled MN pools in the oculomotor nuclear complex (III n.), the vestibulo-ocular projection neuron groups and their axon trajectories (drawn in blue). The position of the stimulating electrode and the location of the contralateral cut used to ensure only ipsilateral stimulation are also shown. **(B)** Black and white image of the CGDA-labeled MN pools seen from the dorsal surface at d10 with positions of ROIs for recording of calcium signals indicated. **(C)** Transverse section through the oculomotor nuclear complex at d10 showing the different MN pools labeled retrogradely with 3kD RDA from the oculomotor nerve on the right side. Note the contralateral location of the SR MN pool. **(D–G)** Sequence of images of the CGDA-labeled MN pools seen from dorsal surface at the indicated stages, showing the migration of SR MNs (red carat) across the midline (dashed line). Scale bars: 100 μm **(B, C)**; 50 μm **(D–G)**.

All the experimental procedures were performed according to EU and NIH regulations and to guidelines established by the Norwegian National Research Animal Care Committee and the chief veterinarian at the animal facility of the Institute of Basic Medical Sciences, University of Oslo. Fertilized White Leghorn chicken eggs (Lohman race, Samvirke Kylling, Norway) were incubated at 38–39°C in a forced draft incubator for 6–11 days, at which time the embryos were removed from the eggs, immediately anesthetized by immersing in ice-cold artificial cerebrospinal fluid (ACSF, containing 120 mM NaCl, 5 mM KCl, 1.5 mM CaCl_2_, 1.0 mM MgCl_2_, 21 mM NaHCO_3_, 0.58 mM NaH_2_PO_4_, and 15 mM glucose and continuously bubbled with a mixture of 95% O_2_ and 5% CO_2_ to keep pH 7.4) and decapitated. Staging was done using the Hamburger and Hamilton system (40) as well as using whole integer multiples of 24 h as we have used in earlier studies [for example, the stage denoted day 7 (d7) = 7 × 24 h of incubation]. The dorsal aspect of the cranium was removed to expose the brainstem, and facial structures were dissected away to expose the oculomotor nerves. To label oculomotor MNs with the dextran-conjugated calcium indicator, Calcium Green-1 dextran-amine (CGDA, 3kD, Molecular Probes/Invitrogen, USA), one of the oculomotor nerves was exposed and cut near the root, and small crystals of the indicator attached on tips of fine needles were applied to the proximal cut end (see (11) for details). Ten-15 min later the entire brain was dissected out and the optic tecta and cerebellum were removed to expose the bottom of the fourth ventricle and the cerebral aqueduct. Then vestibulo-ocular (VO) axons coursing in the medial longitudinal fascicle (MLF) ipsilateral to the labeled oculomotor nerve were labeled with tetramethylrhodamine dextran amine (RDA, 3kD, Molecular Probes/Invitrogen, USA) by applying RDA to a unilateral cut made in the MLF at the level of the glossopharyngeal nerves. This was done to visualize the vestibulo-ocular axons directly under the fluorescence microscope, facilitating the correct placement of the stimulating electrode (see below). RDA was also applied to the contralateral MLF at the level of the trochlear nuclei (that is, at a more rostral level, indicated by “cut” in Figure 2A) to label vestibulo-ocular projection neuron populations retrogradely (1, 11) so that they could also be used as landmarks under the fluorescence microscope. This also prevented conduction of impulses in the vestibulo-ocular axons coursing in the MLF contralateral to the stimulated side from reaching the oculomotor nuclei. Following the dextran-amine applications, the preparations were incubated for 4–6 h at room temperature (approximately 24°C) until labeling was sufficient for visualization and optical recording.

### Stimulation and optical recording

After being trimmed at the levels of the diencephalon and the upper cervical cord, the preparation was pinned dorsal side up in a recording chamber and placed under an upright fluorescent microscope (Axioskop, Carl Zeiss, Germany) equipped with a 10 × water immersion objective (UMPlanFl, 0.3 NA, Olympus, Japan), fluorescein and rhodamine filter sets, a 0.4 × C-mount adapter lens, a CCD camera (Photometrics Cascade, Roper Scientific, USA) and, as a light source for epi-illumination, a 100 W halogen lamp driven by a regulated DC power supply (PAN35-20A, Kikusui Electronics Corporation, Japan). By using the appropriate filter sets, the labeled vestibulo-ocular neurons and axons could be visualized separately from the labeled oculomotor motoneurons. Using the rhodamine filter, a concentric bipolar stimulating electrode was placed on the MLF ipsilateral to the labeled oculomotor nerve rostral to the iR-VO projection neuron group (indicated by “stim” in Figure 2A) so that the electrode could stimulate ascending axons from the iR-VO, iC-VO and cC-VO vestibulo-ocular neuron groups. After switching to the fluorescein filter set, through which CGDA could be visualized, single pulse stimuli (0.1–0.4 mA, 1 ms) that evoked maximum responses (as determined empirically) were given three times with 10-second intervals, and the calcium responses in the MNs were filmed from the dorsal surface with the CCD camera and an imaging program (MetaMorph 5.0, Universal Imaging Corporation/Molecular Devices, USA) at 20 ms/frame with binning factor 4 (Figure 2B). During optical recording, the recording chamber was continuously perfused with room temperature (24–26°C) ACSF at 2 ml/minute. The working volume of the recording chamber during perfusion was approximately 0.4 ml (the exact volume varied from about 0.35–0.42 ml depending on the size of the meniscus generated by the water immersion objective). For pharmacological experiments, drugs used were dissolved in ACSF and continuously applied through the perfusion system starting at least 15 min before optical recording. D-(-)-2-amino-5-phosphonopentanoic acid (AP5) and 6-cyano-7nitroquioxaline-2,3-dione disodium (CNQX), nicardipine, and caffeine were obtained from Sigma-Aldrich, and (-)-bicuculline methochloride (BIC) from Tocris.

### Offline data analysis

In transverse sections through the oculomotor nucleus at 8d and older, four MN pools can be clearly distinguished on each side, three of which innervate respectively the ipsilateral inferior rectus (IR), medial rectus (MR) and inferior oblique (IO) muscles, and the fourth of which innervates the contralateral superior rectus (SR) muscle (1, 12). Labeling of one oculomotor nerve therefore gives the labeling pattern seen in Figure 2C. Since we recorded calcium transients in the nucleus from the dorsal surface, these four MN pools appeared in three longitudinal clusters, one on the contralateral side containing the SR MN pool, and two on the ipsilateral side, a lateral cluster containing the IR MN pool and a medial cluster containing the MR and IO MN pools together, since these lie at different depths at the same mediolateral level. Thus, by placing elliptical regions of interest (ROIs) on each of these 3 clusters for offline analysis (Figure 2B), we could extract calcium responses from the corresponding MN pools. This should give recordings that are dominated by, but not exclusive to, the respective MN pools, since dendrite arborization creates some anatomical overlap and light scattering creates some optical overlap. With respect to light scattering in the x-y plane, which could give false positive responses in inactive neurons lying near activated neurons, our calcium recordings of spinal MNs in the neonatal mouse indicate that this falls off by 50% and 75% over about 30μm and 55μm, respectively (13). Since these distances are less than the dimensions of the ROIs we used (90μm wide and about 650μm long) for the two ipsilateral ROIs, light scattering in the x-y plane probably does not cause significant contamination of the recorded responses. We had no good approach to deal with light scattering in the z-axis, so we could not determine whether responses recorded from the ipsilateral medial aperture arose from MR or IO MNs or both. Similarly, the lateral column aperture overlapped part of the Edinger-Westphal (EW) nucleus, which is also labeled retrogradely through the oculomotor nerve and lies dorsal to the IR pool. Based on our earlier anatomical studies, however, the parasympathetic EW neurons receive virtually no collaterals at these stages from the vestibulo-ocular axons stimulated here [but see (14)]. It is therefore unlikely that calcium transients recorded over the IR MN pool are contaminated by synaptic responses in the EW neurons.

An additional complicating factor to be considered is the dramatic morphological change that occurs in the oculomotor nucleus in the developmental time window we studied, during which the individual MN pools aggregate into recognizable pools and the SR MNs migrate across the midline to settle on the side contralateral to their axons (1, 15). These events can be seen in Figures 2D–G. On d6 the labeled oculomotor MNs appeared as a regionalized cluster with a lateral and a medial component on the ipsilateral side (Figures 2D, E). Thus, at this stage we only defined lateral and medial ROIs on that side, aligned with the separate regions that could be discerned. On d7, when SR MNs were in the process of migrating to the contralateral side (Figures 2F, G), we placed a third ROI on or near the midline to capture these. From d8 on we always defined the three ROIs as shown in Figure 2B. In preliminary analyses we tried defining four or more smaller regions within the ipsilaterally projecting MNs to see if we could distinguish finer regional heterogeneity, but we saw no obvious differences and thus decided to use the simpler set of three ROIs for the analyses presented here.

Response intensities averaged over all the pixels within the ROIs were calculated frame-by-frame on MetaMorph, saved as text files, and normalized to the first frame (representing the background intensity within the same ROI) using a custom-made program. The normalized average intensities were then imported to Clampfit 9 (Axon Instruments/Molecular Devices, USA), expressed as waveforms, and their peak amplitudes, rise times and half-widths were measured.

For pseudocolor-coded representation, pixel intensities on each frame were normalized to those of the first frame, filtered with a 3 × 3 median filter, and assigned to a rainbow type color index on MetaMorph, with increasing intensity spanning from blue to red.

### Statistics

Differences of data distributions were tested using the Wilcoxon paired signed rank test for comparisons of responses within a preparation (before and after a manipulation, or comparing different ROIs), the Mann-Whitney U-test for comparisons between two preparations, and the Kruskal-Wallis test with Bonferroni correction for comparisons across multiple developmental stages. In each case significance level was set at *p* < 0.05 (lower significance levels indicated when attained). In graphical presentations of data, bars represent means and error bars represent standard deviations.

## Results

### Basic properties of the synaptic responses

**Figure 3:**
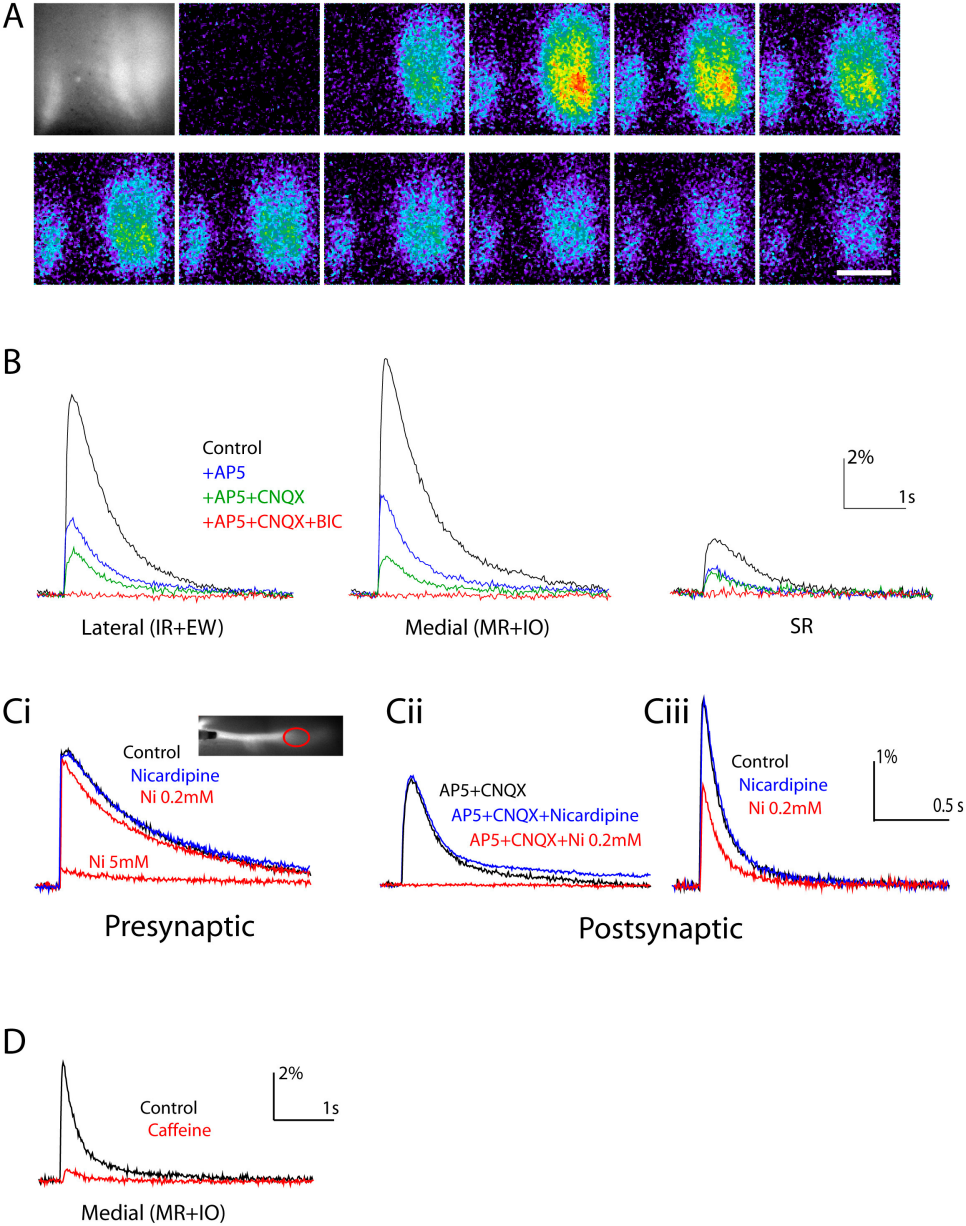
Pharmacological discrimination of different contributions to presynaptic and postsynaptic calcium responses. **(A)** Representative series of video images during postsynaptic response to stimulation of the presynaptic vestibulo-ocular axons. First panel shows a black and white image of the CGDA-labeled MN pools seen from the dorsal surface. Subsequent panels (left to right top row, followed by left to right bottom row) show false color images of CGDA fluorescence taken at 120 msec intervals starting just before a single stimulus pulse, with fluorescence intensity relative to background color-coded on a scale from low intensity (blue) to high intensity (red). CGDA fluorescence increases rapidly and falls more slowly. **(B)** Response waveforms generated from video image series in the indicated ROIs, color-coded according to the indicated superfusion conditions. **(Ci)** Waveforms of calcium signals recorded from the presynaptic axons color-coded according to the indicated superfusion conditions. Inset shows a black and white image of the labeled axons with the stimulation electrode visible to the left, and the ROI used to record the presynaptic signals (red oval). **(Cii, Ciii)** Response waveforms recorded in the lateral ROI, color-coded according to the indicated superfusion conditions. **(D)** Response waveforms recorded in the medial ROI color-coded according to the indicated superfusion conditions. Scale bars: 200 μm **(A)**; fluorescence intensity change in % and time in seconds (s) **(B–D)**.

A typical calcium response in the MNs within the oculomotor (OM) nucleus following stimulation of the MLF is shown for a d9 preparation in Figure 3A. Shortly after a single 1 msec pulse stimulus the entire retrogradely labeled region of the OM nucleus showed a rapid increase in CGDA fluorescence that reached a peak intensity and then faded away. To analyze the response quantitatively, we defined three ROIs positioned over specific MN pools and expressed the average signals from these as waveforms (see Materials and Methods) (Figure 2B). All three ROIs exhibited monophasic calcium elevations lasting for several seconds after each stimulus. The average amplitudes, rise times, and half-widths at d9 were respectively 12.1% ± 2.5% (percent over background), 210 ± 41 ms and 810 ± 227 ms for the lateral ipsilateral ROI (covering the inferior rectus (IR) MNs and parasympathetic MNs of the Edinger-Westphal nucleus), 10.7% ± 2.5%, 234 ± 99 ms and 685 ± 186 ms for the medial ipsilateral ROI (covering the medial rectus (MR) and inferior oblique (IO) MNs), and 2.9% ± 1.3%, 418 ± 134 and 1,127 ± 395 ms for the contralateral ROI (covering the superior rectus (SR) MNs; *n* = 19). The amplitudes and rise times in the contralateral ROI were significantly lower and longer, respectively, than those in the two ipsilateral ROIs (Wilcoxon paired sign rank test for contralateral vs. lateral ipsilateral ROI, z = −3.82 and z = −3.22, respectively, *p* < 0.005). Cutting the MLF between the stimulating electrode and the OM nucleus eliminated the responses (*n* = 7 preparations, ranging from d7-d10), indicating that they were mediated by axons coursing in the stimulated MLF.

#### Influence of indirect pathways

Although monosynaptic connections from VO projection neurons to OM MNs constitute the main pathways of the vertical and rotational components of the VOR, additional polysynaptic connections exist (16). For example, excitatory inputs to the MR MNs originate from interneurons in the abducens nucleus (“abducens INs”), which constitute the second relay in a pathway from VO projection neurons to LR MNs, mediating the horizontal component of the VOR (reviewed in 3). The abducens IN axons cross the midline to ascend in the MLF to the contralateral MR MN pool. The abducens INs are on hodological grounds included in the cC-VO group, which also contains VO projection neurons that project to the contralateral SR and IO MNs. Thus, the abducens IN axons are stimulated together with cC-VO, iC-VO, and iR-VO axons from the stimulation site in the MLF, because this site is rostral to where the cC-VO/abducens IN axons cross the midline. Thus, our MLF stimulation engages monosynaptic connections to OM MNs from cC-VO/abducens IN axons, iC-VO axons, and iR-VO axons (cR-VO axons cross the midline rostral to the stimulation site, and therefore are not included; Figure 2).

An additional polysynaptic pathway that could have been activated from our stimulation site is that through the interstitial nucleus of Cajal (INC) and the rostral interstitial nucleus of the MLF (riMLF). Some VO axons are known to have collaterals projecting to these nuclei, which in turn project caudally to the OM nucleus (17–21). Consistent with this, in the chicken embryo we showed previously that VO axons extend in the MLF beyond the OM nucleus to at least the level of the INC, in parallel with INC and riMLF axons that descend in the MLF to the OM (41). To assess whether this pathway contributes to the response, we made an incision between the OM nucleus and the INC to sever both the VO axons ascending to the INC and the descending INC and riMLF axons and compared the response before and after. Although the effects varied somewhat among individual preparations, there were on average no significant differences (*n* = 7, d9-11; Wilcoxon signed rank test, z = −0.59, *p* > 0.05). We conclude that this indirect polysynaptic pathway does not contribute significantly to the responses we recorded. It may not be fully established at the embryonic stages used here.

#### Mechanisms underlying the calcium responses

##### Neurotransmitters involved

We next considered the mechanisms involved in generating the calcium responses. We first examined the effects of fast neurotransmitter receptor blockers. Superfusion of preparations with 50 μM AP5 (an NMDA receptor blocker) and 10 μM CNQX (an AMPA receptor blocker) reduced the responses in each of the three ROIs in an additive manner, and the remaining component was eliminated by 20 μM bicucculine (BIC, a GABA_A_ receptor blocker) (*n* = 35 preparations, d7-11; Figure 2B). Thus, measurable responses were mediated by these receptors (NMDARs, AMPARs, and GABA_A_Rs, respectively) and evidently no others. This result also suggests that the activation of GABA_A_Rs on the OM MNs is depolarizing at these developmental stages, since a hyperpolarization would not be expected to elicit a calcium response (22, 23).

##### Voltage gated calcium channels and calcium-induced calcium release

Calcium elevation arising from the activation of synaptic inputs could involve several mechanisms, including influx through calcium-permeable ionotropic receptors such as NMDARs and calcium-permeable AMPARs, indirect activation of voltage-gated calcium channels (VGCCs) by depolarizing postsynaptic potentials or action potentials, IP_3_-induced calcium release via metabotropic receptors, and calcium-induced calcium release (CICR) [reviewed in Augustine et al. (24) and Hartmann and Konnerth (25)]. A contribution to calcium influx through NMDARs was already demonstrated, since blockade of NMDARs diminished the response by more than half (Figure 3B), and a contribution from IP_3_-induced calcium release via metabotropic receptors was already excluded, since simultaneous blockade of NMDARs, AMPARs, and GABA_A_Rs extinguished the response (see above).

To assess which VGCCs might be involved, we examined the effects of two VGCC blockers, nicardipine (a blocker specific to L-type calcium channels) and Ni^2+^ (a non-specific calcium channel blocker). We first tested whether these blockers affected the presynaptic release of neurotransmitter, by labeling the VO axons in the MLF anterogradely with CGDA and recording calcium transients in the axon terminals *en masse* (Figure 3Ci “presynaptic”). As expected, since presynaptic calcium channels are primarily N- and P/Q-type, nicardipine had no effect on the presynaptic calcium transient. Ni^2+^ on the other hand exerted a nearly complete block at 5 mM, but hardly any block at 0.2 mM (*n* = 4 preparations, d9 and 10; Figure 3Ci “presynaptic”). This indicates that the presynaptic calcium elevation is mediated by Ni^2+^-sensitive, non-L-type calcium channels (presumably N or P/Q channels) and is maintained at the lower Ni^2+^ concentration.

We then assessed the effects of nicardipine and 0.2 mM Ni^2+^ on the postsynaptic responses. Since NMDARs are calcium permeable and AMPARs can be calcium permeable but GABA_A_Rs are not, we first examined the effect of these blockers on the residual GABA_A_R-mediated component in the presence of AP5 and CNQX. Whereas 10 μM of nicardipine had no effect, 0.2 mM Ni^2+^ eliminated the GABA_A_R-mediated response entirely (*n* = 3 preparations, d9 and 10; Figure 3Cii “postsynaptic”). This suggests that the GABA_A_R-mediated response involves a depolarization that activates Ni^2+^-sensitive, non-L-type calcium channels (presumably R- or T-type channels). We then tested the effect of the blockers under control conditions, in the absence of AP5, CNQX and BIC. The aggregate postsynaptic calcium response was only partially reduced by 0.2 mM Ni^2+^ (by 48.1 ± 2.7% in lateral ipsilateral, 44.2 ± 0.9% in medial ipsilateral and 46.3 ± 19.3% in SR ROIs, *n* = 3 preparations, d9, Figure 3Ciii, “postsynaptic”), consistent with a substantial proportion of the aggregate response deriving from calcium influx through NMDARs and/or AMPARs. However, on the basis of these experiments we cannot rule out a contribution from Ni^2+^-insensitive VGCCs specifically activated by glutamate receptor-mediated depolarization and different from the VGCCs activated by GABA_A_R-mediated depolarization (N-type channels, for example).

We also examined the effect of caffeine, a sensitizer of calcium-induced calcium release (CICR) from intracellular stores. This was only done at d9 and d10. Continuous perfusion with 10 mM caffeine elicited varying calcium responses in the OM MNs, including relatively small, gradual calcium elevations and relatively larger, more abrupt calcium elevations along with calcium spikes. These responses to caffeine alone had largely disappeared by about 30 min after the start of exposure. At this time we stimulated the MLF, and the aggregate responses were markedly reduced relative to control in all 3 ROIs (by 61.3% ± 26.8% in lateral ipsilateral, *n* = 3; by 68.1 ± 17.9% in medial ipsilateral, *n* = 3; and by 59.2% in SR, *n* = 1 Figure 3D). This suggests a substantial contribution from CICR to the aggregate response at d9 and d10, that is lacking after intracellular stores of calcium are exhausted by the caffeine stimulation.

Altogether, we conclude that the calcium elevations that underly the synaptic responses to VO and abducens IN axon stimulation arise by virtue of (1) calcium influx through NMDARs and/or calcium-permeable AMPARs, (2) calcium influx through VGCCs activated by GABA_A_Rs and probably also by the glutamate receptors, and (3) at least at later stages (d9 and d10) through CICR from intracellular stores.

### Development of synaptic responses

#### First overt response observed at d7

**Figure 4:**
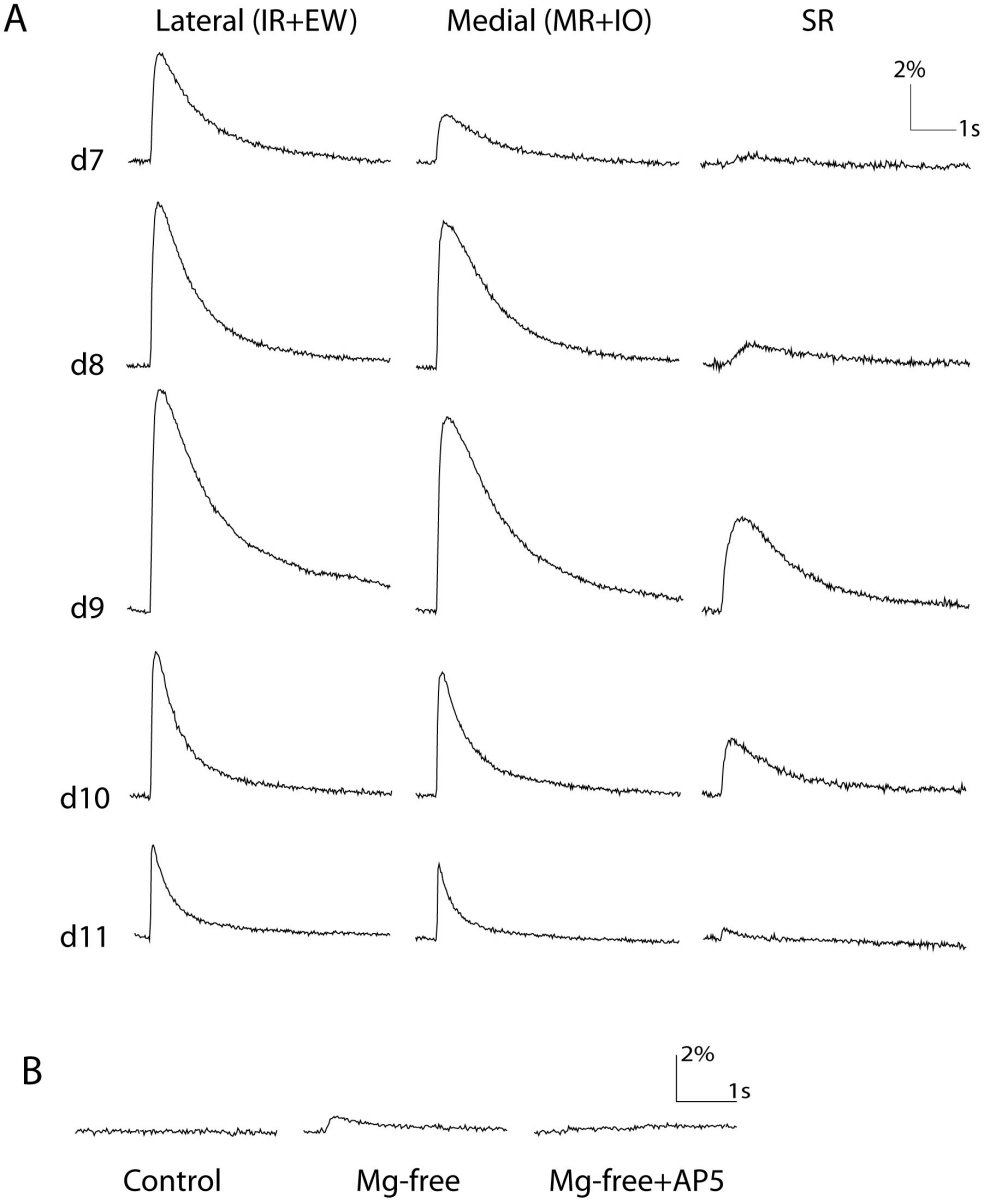
Development profile of postsynaptic response amplitudes. **(A)** Total response amplitude increased from d7-d9 and then decreased from d9-d11 in all three ROIs. **(B)** In d7 preparations that initially did not exhibit a response (see Table 2), removal of Mg^2+^ from the superfused ACSF revealed an AP5-sensitive (NMDAR-mediated) response. Scale bars indicate fluorescence intensity change relative to background in % and time in sec (s).

We next asked how the responses developed as synaptic connections are formed between the VO afferents and the OM MNs. In a previous study we showed that VO axons in the MLF reach the level of the OM nucleus on d5 but do not begin to sprout terminal collaterals until about d6.5 (1, 26). Consistently, no response was evoked by stimulation of VO axons in the MLF on d6 (Table 1). The earliest responses appeared on d7 in the ipsilateral lateral and medial ROIs in 50% of preparations and in the SR ROI in 10% of preparations (Figure 4A, Table 1).

**Table 1:** Proportions of preparations showing a response at the indicated stages.

	**Lateral**	**Medial**	**SR**	***n***
d6	0%	0%	0%	3
d7	71%	65%	12%	17
d8	100%	100%	100%	6
d9	100%	100%	89%	19
d10	100%	100%	73%	11
d11	100%	100%	45%	11

#### Initially silent synapses mediated by NMDARs

At early stages of synaptogenesis, it has been shown in several systems that many glutamatergic synapses activate only NMDARs, such that their functional effect is masked by extant extracellular Mg^2+^ in a voltage-dependent manner (thus called “silent synapses”) (37, 38). To check whether this might be the case here, we replaced the perfusate with Mg^2+^-free ACSF in the d6 and d7 preparations that did not exhibit any responses in normal ACSF. In four out of five d7 preparations that did not show responses in normal ACSF, this revealed small responses in the ipsilateral lateral and medial ROIs that were suppressed by AP5 (Figure 4B, Table 2). No such responses could be revealed by Mg^2+^-free ACSF in d6 preparations (*n* = 3 preparations). This suggests that the earliest potentially functional synapses from VO axons onto OM MNs arise early on d7 and activate predominantly NMDARs.

**Table 2:** Earliest synaptic responses revealed by Mg^2+^-free saline in d7 preparations lacking response in normal saline.

	**HH stage**	**Normal**			**Mg**+**2–free**		
		**Lateral**	**Medial**	**SR**	**Lateral**	**Medial**	**SR**
Prep 1	30	–	–	–	+	+	–
Prep 2	30	+	–	–	+	+	–
Prep 3	29	–	–	–	–	–	–
Prep 4	29	–	–	–	+	–	–

#### Response amplitudes increase until d9 and decline thereafter

As development proceeded from d7 to d9, the synaptic response amplitudes increased progressively, and then fell more steeply from d9 to d11 (Figure 4A, Table 3). This was consistent among ROIs: amplitudes increased significantly from d7 to d9 by about 45% in the lateral ROI and 100% in the medial ROI, and then fell significantly from d9 to d11 by about 50% in each (Table 3). Responses in the SR ROI were always smallest but exhibited the same tendency (not analyzed statistically). Rise times and half-peak widths underwent developmental changes as well. There was an overall decrease in rise time from d7 to d11 by about 60% in the lateral and medial ROIs, and an overall decrease in half-peak width from d7 to d11 in the same ROIs by about 45%. In both cases most of the decrease occurred from d9 to d11, paralleling the decrease in response amplitude (Figure 4A, Table 3). Responses in the SR ROI had substantially longer rise times than those in the other regions, which might reflect a more varied conduction velocity in the VO axon collaterals that innervate the SR MNs (see SR ROI traces in Figures 3B, 4).

**Table 3:** Developmental dynamics of calcium responses.

**ROI**	**d7**	**d8**	**d9**	**d10**	**d11**
	***n** =* **6**	***n** =* **5**	***n** =* **19**	***n** =* **8**	***n** =* **5**
**Lateral (IR** + **EW)**					
Amplitude	8.41 ± 1.38	11.12 ± 0.7 (↑^*^)	12.06 ± 2.47 (↑^**^)	8.22 ± 1.77 (↓^*^)	6.52 ± 1,48 (↓^**^)
Rise time	272 ± 43	176 ± 49 (↓^*^)	210 ± 41	131 ± 36 (↓^*^)	108 ± 22 (↓^**^)
Half width	804 ± 124	729 ± 104	810 ± 227	572 ± 105 (↓^*^)	432 ± 71 (↓^*^)
**Medial (MR** + **IO)**					
Amplitude	5.17 ± 1.09	8.63 ± 1.36 (↑^*^)	10.70 ± 2.51 (↑^**^)	6.93 ± 1.40 (↓^*^)	5.53 ± 1.18 (↓^*^)
Rise time	315 ± 68	226 ± 106	234 ± 99	144 ± 63 (**↓**)	113 ± 22 (↓^**^)
Half width	647 ± 151	615 ± 127	685 ± 186	526 ± 97 (↓^*^)	385 ± 89 (↓^*^)

Amplitudes are shown in fractional increase in fluorescence intensity over background, rise times and half widths are shown in msec, and values indicate means and standard deviations. Sample sizes (n) indicate number of preparations. Arrows indicate direction of change relative to the prior developmental stage. Single asterisks indicate statistically significant changes relative to the prior developmental stage, and double asterisks represent statistically significant changes relative to the next earlier developmental stage (for example d11 relative to d9). Both ^*^ and ^**^ indicate *p* < 0.01 (Kruskal-Wallis test of independent samples with Bonferroni correction for multiple tests). Aside from a dip in rise time at d8 in the lateral ROI which is not seen in the medial ROI, all changes are consistent in the two ROIs.

#### Development of NMDAR, AMPAR, GABA_*A*_R components

**Figure 5:**
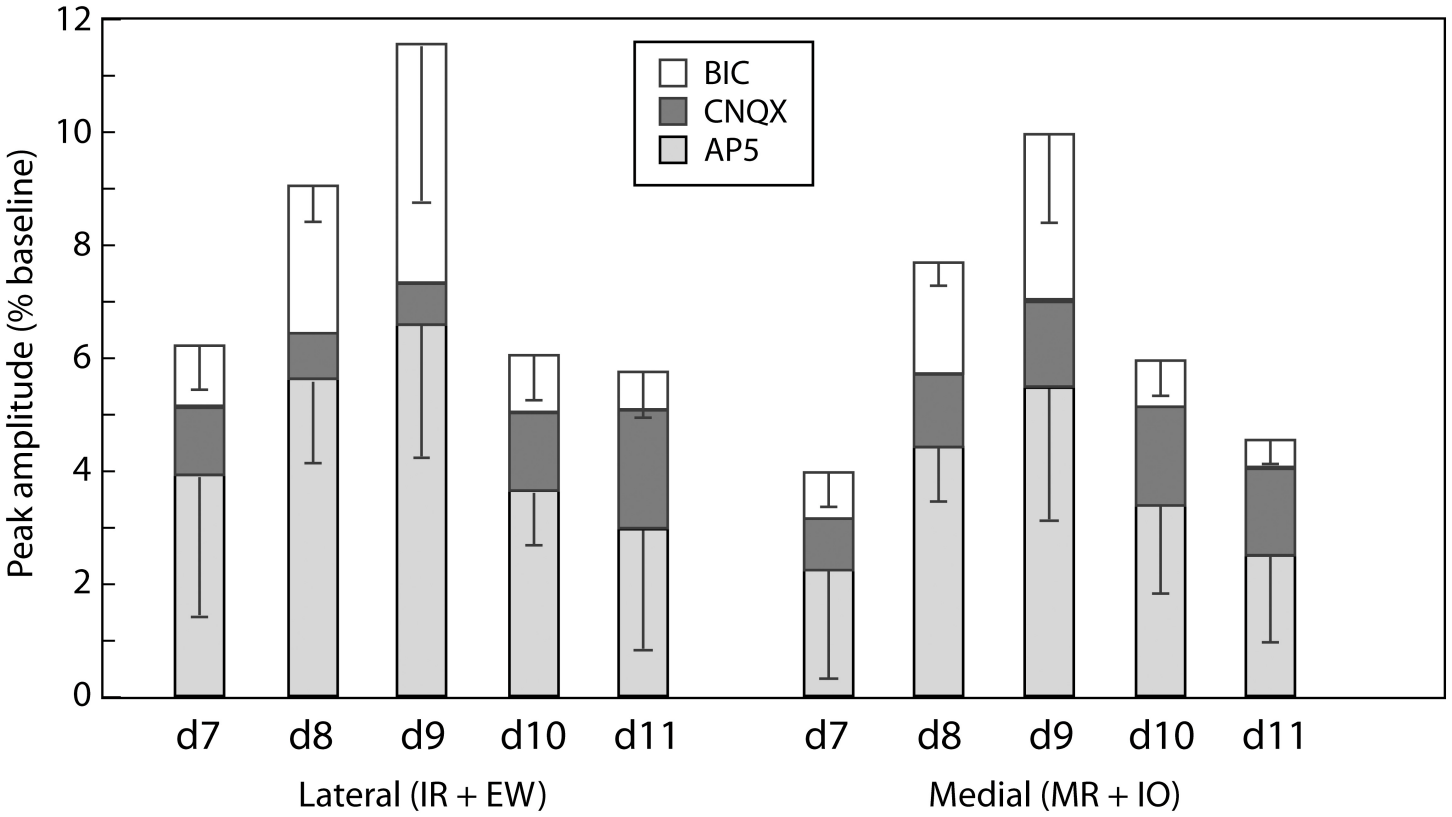
Quantitative developmental profile of GABAergic and glutamatergic components. Shown are means (bar heights) and standard deviations (error bars) for the indicated response components at the different developmental stages (data only from those preparations exhibiting responses, see Table 1). Error bars for the CNQX-sensitive (AMPAR-mediated) components are not shown for visual clarity but are similar or smaller than those shown for the BIC-sensitive (GABAR-mediated) components. NMDAR-mediated (AP5-sensitive) components tended to be largest throughout the developmental period studied.

We then asked how the contributions of the 3 receptor types, NMDARs, AMPARs, and GABA_A_Rs, changed during this same developmental period (Figure 5). To do this, we sequentially applied 50μM AP5, 10μM CNQX, and 20μM BIC, assessed the reductions in peak amplitudes caused by the addition of each drug, and regarded the reductions as the amplitudes of NMDAR-, AMPAR- and GABA_A_R-mediated components, respectively. We limited this analysis to the ipsilateral lateral and medial ROIs because the responses in the SR ROI were so small that often they fell below the noise level (0.5%) already after the AP5 application and it was impossible to evaluate the other components.

From d7, when overt responses first appeared, until d11, the latest stage studied, all ipsilateral lateral and medial ROI responses consisted of all 3 components (*n* = 6, 5, 8, 4, and 5 preparations on d7-d11 respectively, Figure 5). The following trends (some of which reached statistical significance) are evident when considering the relative contributions of these components. The NMDAR-mediated component was always the largest throughout the developmental period, contributing on average more than half the total amplitude (*p* < 0.01, Kruskal-Wallis test with Bonferoni correction). The GABA_A_R-mediated component on average was the second largest at d8 and d9, but diminished substantially by d11 (*p* < 0.05, Kruskal-Wallis test with Bonferoni correction). The AMPAR-mediated component was relatively smaller and did not change significantly from d7-d11.

We did not perform a systematic permutation of the order of antagonist application. This is primarily because when BIC was applied first, the effect was complex and depended on the developmental stage (see below).

#### Three phases in the development of functional connections

From these results, there seem to be three phases in the development of the synaptic responses in the OM MNs. The first phase corresponds to the half-day period early on d7 during which only covert responses can be elicited via NMDARs in the absence of Mg^2+^. The second phase corresponds to the subsequent 2.5-day period through d9, during which the total response increases in amplitude and the NMDAR- and GABA_A_R-mediated components dominate. The third phase corresponds to the subsequent 2 days, during which the total response amplitude declines, and the proportional contribution of the GABA_A_R-mediated component decreases.

#### Inhibitory effect of GABA_*A*_R-mediated input on glutamatergic inputs

There are many possible explanations for the response decline during the third phase (see Discussion). One is that GABA_A_R-mediated transmission might switch from excitatory to inhibitory. To see if this pertained, we applied 20 μM BIC alone to d9 and d11 preparations prior to stimulation of the presynaptic axons to block the GABAergic input. At both d9 and d11, responses in the ipsilateral lateral and medial ROIs were enhanced in the presence of BIC in most cases (5/6 preparations in lateral ROI, 4/6 preparations in medial ROI), indicating a predominantly inhibitory effect of GABA. The degree of enhancement, when it occurred, was significantly different from control (Table 4). Thus, the GABAergic input, despite generating calcium signals, was *de facto* inhibitory in most cases at both time points. The decrease in total response amplitude from d9 to d11 therefore cannot be attributed solely to a switch from GABAergic excitation to GABAergic inhibition, since GABAergic inhibition is already evident at d9.

**Table 4:** Effect of bicucculine (BIC) alone on responses.

**ROI**	**Control**	**20 μM BIC**	**Recovery**
**Lateral (IR**+**EW)**			
Enhanced (d9 *n =* 2, d11 *n =* 3)	1.00	1.30 ± 0.32 (*n =* 15)^*^	1.04 ± 0.12
Diminished (d11 *n =* 1)	1.00	0.58 ± 0.01 (*n =* 3)	1.02 ± 0.21
Aggregate (d9 *n =* 2, d11 *n =* 4)	1.00	1.18 ± 0.32 (*n =* 18)	1.04 ± 0.13
**Medial (MR**+**IO)**			
Enhanced (d9 *n =* 2, d11 *n =* 2)	1.00	1.39 ± 0.11 (*n =* 12)^*^	0.99 ± 0.14
Diminished (d11 *n =* 2)	1.00	0.44 ± 0.23 (*n =* 6)	0.94 ± 0.12
Aggregate (d9 *n =* 2, d11 *n =* 4)	1.00	1.07 ± 0.48 (*n =* 18)	0.97 ± 0.13

All values are normalized to control values. Three exposures and recordings were made in each preparation. Values thus represent the averages and standard deviations of 3x the indicated number of preparations. Differences between before and after exposure to BIC were not significant (*p* < 0.1) when both enhanced and diminished responses were included (aggregate response), but reached *p* < 0.005 (^*^) when only enhanced responses were included (Wilcoxon signed rank test, z= −3.41 for lateral ROI, z= −3.06 for medial ROI).

#### Paired-pulse and train stimulation

To obtain further clues about the cause of the developmental decline in response, we used paired-pulse stimulation in d9 and d11 preparations. Paired-pulse facilitation (PPF) is often used to assess the probability of transmitter release (Pr) from presynaptic terminals (27). Greater PPF is usually taken to indicate a low initial Pr, although this relationship can vary widely at some synapses (28). PPF is measured as the ratio of the second EPSP to the first EPSP after two successive stimulating pulses are applied to the presynaptic axons with a brief interval (typically within a few 100 milliseconds). Here, we did not record EPSPs, but postsynaptic calcium responses. Since a facilitation of the calcium response could reflect changes in a number of downstream processes between the EPSP and calcium elevation, it does not necessarily indicate a presynaptic change. Nevertheless, it can provide information that can rule out certain trivial explanations for the developmental decline (see Discussion).

Since it was impossible to resolve responses to each of paired pulses (which we separated by 50 msec) due to the slow kinetics of the calcium response, we instead applied a single-pulse stimulus and then 10 seconds later paired-pulse stimuli (with a 50 msec interval) and calculated the ratio of the peak calcium response to the paired-pulse stimuli to the peak response to the single-pulse stimulus. This assumes that the first of the paired-pulse stimuli gives the same response as the preceding single-pulse stimulus, and that the peak amplitude of the response to paired-pulse stimuli accurately reflects the enhancement above a single pulse. The first assumption appeared to be met in our experiments, since single pulses with 10 second intervals gave constant responses (data not shown). The second assumption relies on the absence of non-linear summation in the processes leading up to the calcium elevation. This possibility was deemed too complicated to be surveyed here due to the multifactorial nature of the calcium elevation.

**Figure 6:**
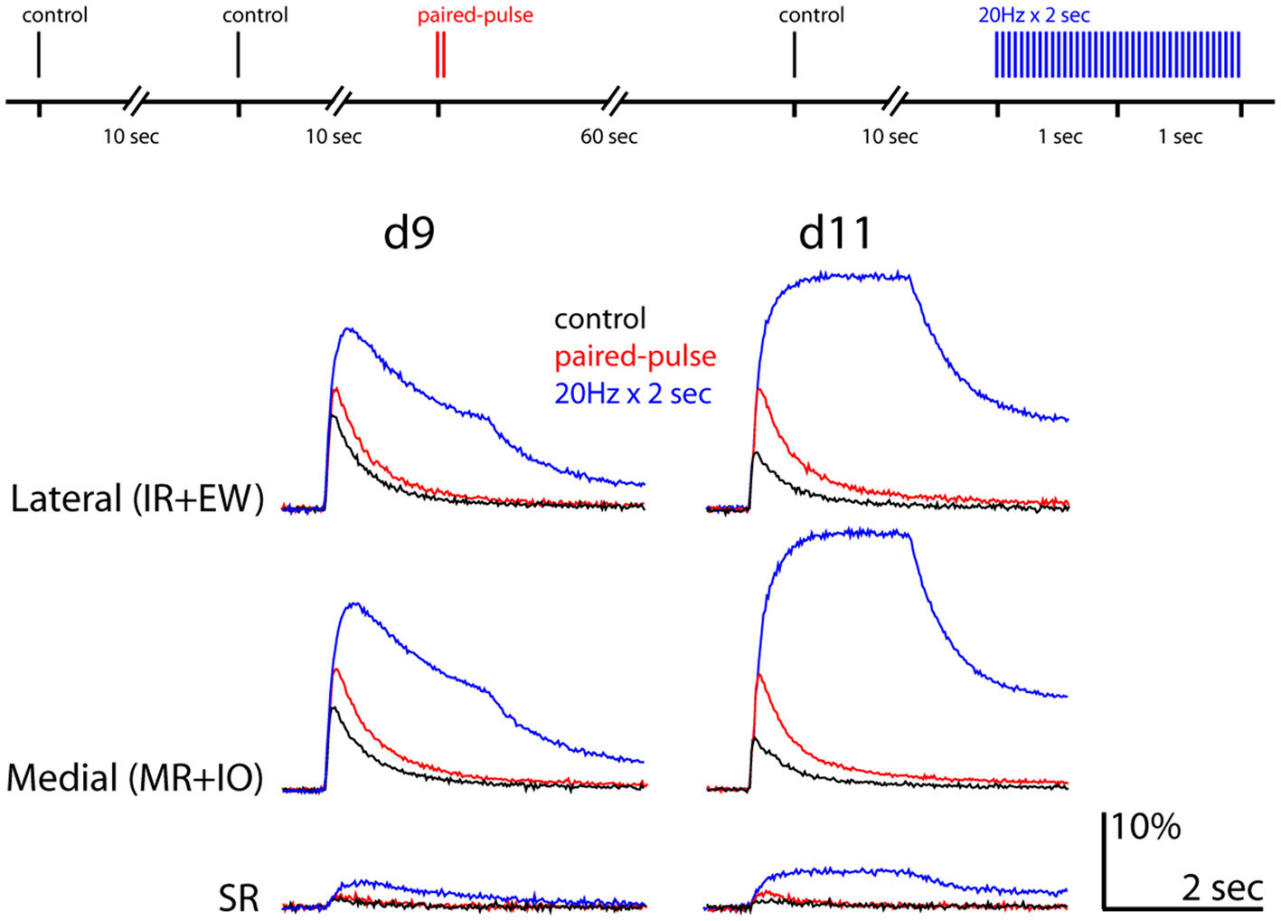
Paired pulse and train stimulation demonstrate facilitation of the postsynaptic responses. Experimental procedure shown at top. Response waveforms shown at d9 and d11 in each ROI color-coded according to the indicated stimulation conditions (train stimulation was 20 Hz for 2 sec). Scale bars indicate fluorescence intensity change relative to background in % and time in sec.

Whereas d9 preparations exhibited relatively little PPF (118.7 ± 13.0% of control in the lateral ipsilateral ROI, 138.2 ± 12.5 of control in the medial ipsilateral ROI; *n* = 6), d11 preparations showed substantial PPF (218.5 ± 31.6% of control in the lateral ipsilateral ROI, 216.7 ± 18.3% of control in the medial ipsilateral ROI; *n* = 5; Figure 6). To determine if the responses to the paired-pulse stimuli may have saturated, we also applied train stimulation (20 Hz for 2 sec) and found that this facilitated responses on both d9 and d11 to much greater levels than obtained with paired-pulse stimulation (Figure 6). Thus, the responses to the paired-pulse stimulation were not saturated at either stage. Another striking feature of the responses to train stimulation was that whereas the response on d9 started to decline in the middle of the train, the response on d11 maintained a plateau.

## Discussion

### Brief summary of results

As far as we know, this is the first study that has characterized physiologically the embryonic development of defined excitatory and inhibitory inputs onto extraocular MNs. We have addressed this issue in the specific context of inputs from VO projection neurons (and abducens INs) whose axons course in the MLF and terminate on MN targets in the oculomotor (OM) nucleus. This is not an exclusive relationship; the VO axons in question do not innervate only OM MNs (they also innervate MNs in the trochlear nucleus), nor do the OM MNs only receive inputs from these axons (they also receive inputs from cR-VO axons and from non-VO sources). For this reason our results may not be generally representative of the development of synapses on extraocular MN populations or even on these specific OM MN populations. Moreover, our results cannot be directly compared to information obtained using techniques that assess inputs from all sources indiscriminately, for example through recording of spontaneous postsynaptic potentials or through characterizing synaptic profiles ultrastructurally in the absence of any tracing. The advantage of our study is that the system of VO neuron inputs and MN targets has a functional coherence within the system of excitatory and inhibitory synaptic connections that creates coordinated patterns of MN activity within the well-defined vestibulo-ocular reflex (VOR). From the perspective of the emergence of network function it seems therefore more relevant to ask how particular inputs such as these develop than to assess wholesale the development of all the diverse synaptic inputs impinging on MNs.

We have shown that glutamatergic and GABAergic synaptic inputs are functional within hours after the first contact of OM MNs by VO axon terminals (contact as assessed anatomically) (1). We show further that the very first synapses to form that can be revealed physiologically are glutamatergic, acting through NMDARs, but these are functionally silent unless the Mg^2+^ block of NMDARs is removed. Thereafter, within a day of first contact, AMPAR-mediated glutamatergic and GABA_A_R-mediated GABAergic synapses become functional, and all three receptor-specific inputs lead to postsynaptic calcium transients with a similar waveform. As development proceeds, responses mediated by all three receptor types increase in magnitude up to d9, after which they diminish in magnitude. In parallel, the relative contributions from the different receptors change, with NMDAR- and GABA_A_R-mediated responses dominating during the increase and GABA_A_R-mediated inputs diminishing proportionally during the decline. In keeping with the dynamics of calcium signals, the durations of the total (aggregate glutamatergic and GABAergic) responses are on the order of several seconds, with half-peak durations of 1–2 sec, depending on stage. Paired-pulse stimulation experiments show a clear facilitation (PPF) that increases from d9 to d11. This is compatible with, but does not provide definitive evidence for, a decrease in the probability of neurotransmitter release (Pr) during the developmental period when response magnitudes diminish.

### The hodological organization of the synaptic connections and the special case of responses in SR MNs

The ipsilateral lateral and medial ROIs register responses respectively in the IR+EW and MR+IO MN pools. Based on the site of the stimulation electrode, specific inputs as shown in Figure 1 are expected to be activated. These are excitatory and inhibitory inputs to the IR MN pool from respectively the cC-VO group and iR-VO group, excitatory input to the MR MN pool from the abducens INs, and inhibitory input to the IO MN pool from the iC-VO group. Thus, the expected pattern of activated excitatory and inhibitory inputs is consistent with what we recorded in the ipsilateral lateral ROI (glutamatergic and GABAergic inputs to the IR MN pool) and the ipsilateral medial ROI (glutamatergic input to the MR MN pool and GABAergic input to the IO MN pool). The input that is missing is the excitatory input to the IO MN pool, which derives from the cR-VO group, which was not stimulated as its axons do not project in the MLF.

It is unclear which connections are involved in the responses recorded in the contralateral SR ROI. The contralateral SR MN pool receives excitatory and inhibitory inputs from respectively the cR-VO group ipsilateral to the stimulation site and the iC-VO group contralateral to the stimulation site, neither of which were stimulated here. The responses in the contralateral SR MN pool were both glutamatergic and GABAergic but distinctly lower in amplitude than those in the other MN pools, and not consistently present. One possibility is that current spread from the stimulating electrode could have engaged a few excitatory axons from the cR-VO group ipsilateral to the stimulation site as they cross the midline despite their location rostral to the stimulation site (Figure 2A). This could have given rise to a glutamatergic response in the contralateral SR MN pool. Similar current spread to inhibitory iC-VO axons in the contralateral MLF, which could have given rise to a GABAergic response, is ruled out by the cut we made in the contralateral MLF (Figure 2A). Alternative possibilities include connections mediated by commissural collaterals from the axons stimulated, and electrical coupling between SR MNs on the two sides. Further investigation will be needed to determine what underlies these responses, and whether they represent a phenomenon (such as commissural collaterals or electrical coupling) that might be relevant to the other MN pools as well.

### Technical considerations

The advantage of calcium imaging is that it provides a rapid optical assessment of responses in large numbers of neurons simultaneously. As a method for assessing functional synaptic connectivity, calcium imaging does have limitations, however. It does not provide information about membrane potential changes, it has low temporal resolution, and it does not readily reveal inhibition in the absence of ongoing excitation. Moreover, calcium signals are the product of potentially diverse cellular processes with different kinetics and spatial organization, and are therefore difficult to relate directly to postsynaptic potentials. Nevertheless, calcium imaging can provide a clear indication that synapses are indeed functional, and through combination with pharmacological manipulation can reveal which receptors, channels and other calcium metabolic mechanisms are involved. This notwithstanding, it only reveals inputs that generate calcium transients, and is therefore contingent on the presence of calcium permeable neurotransmitter receptor-channel complexes, of voltage-gated calcium channels that can be opened by postsynaptic potentials, or of calcium pumps whose activity is altered by synaptic activity.

### The nature of the glutamatergic responses

AMPAR-mediated responses were present throughout the period of development studied, indicating the presence of Ca^2+^-permeable AMPARs, which would imply expression of the GluR2 subunit (29). As far as we know there is no information about expression of the different AMPAR subunits in the OM MNs of the chicken embryo at the stages we have studied here, but there is incidental observation of Glu1R, Glu2R, and Glu3/4R expression in the dorsolateral region abutting the EW nucleus starting around d14 (30). The Glu2R subunit is known to be expressed by a small minority (about 2%) of EW neurons in the d12 chicken embryo, increasing to about 15% in hatchling chicks (30, 31). The Glu2R subunit is also expressed in adult rodent and human OM MNs (32, 33).

Some additional information is available from studies of resistance to the disease amyotrophic lateral sclerosis (ALS) in OM MNs. This involves site-specific Glu2R pre-mRNA editing and *lower* expression of Glu2Rs in OM MNs relative to most skeletal MNs in rodents and humans (34, 35). Thus, the pronounced AMPAR-mediated calcium responses in OM MNs in the chicken embryo could be a stage-dependent property, wherein Glu2R subunit expression is high at embryonic stages but diminishes later, or it could reflect a species-specific property, in which mammalian OM MNs have low Glu2R expression (and thus are resistant to ALS) whereas avian OM MNs have high Glu2R expression. These possibilities can be tested in future studies and could shed light on the development and evolution of ALS susceptibility and resistance.

Calcium responses mediated by NMDARs are to be expected, since NMDARs are by nature permeable to calcium. Here we found that the NMDAR-mediated component is proportionally the largest throughout the developmental period studied, indicating potentially a prominent role of synaptic plasticity in the VO-to-OM MN connection during development. NMDARs are known to be expressed in the OM MNs of adult rats, and here again there are differences among MN populations related to ALS-resistance (36).

We did not test directly whether the AMPAR- and NMDAR-mediated responses also involve activation of VGCCs (by applying VGCC blockers in the presence of BIC), but this is likely given the presumed depolarizing nature of the glutamatergic inputs. In addition, it appears that at least at later stages CICR is a contributing factor, since prior forced release of calcium from intracellular stores by caffeine substantially reduces the synaptically induced calcium responses.

### The nature of the GABAergic response

We show that the GABA_A_R-mediated calcium responses involve Ni^2+^-sensitive, non-L-type VGCCs, consistent with a depolarizing action of GABA. In preliminary experiments using patch clamp recording we have observed directly that the GABA_A_R-mediated responses are depolarizing. However, we have not assessed reversal potentials and therefore cannot conclude whether the GABA_A_R-mediated depolarization is truly excitatory. The increase in response in most preparations when the GABAergic input was blocked by BIC suggests rather that this input is predominantly inhibitory (at least at the stages tested, d9 and d11). This could occur if the associated reversal potential lies below threshold. In this case GABA_A_R activation would shunt the glutamate-mediated depolarization, leading to a smaller aggregate response, despite the fact that GABA_A_R activation elicits a calcium influx through VGCCs that can summate with glutamate-elicited calcium influx. Nevertheless, the few cases in which responses decreased in the presence of BIC suggest that the GABA_A_R-mediated depolarization may be transitioning from excitatory to inhibitory during this developmental period, but to varying degrees in different embryos.

### Time course of functional synaptic development

We show that glutamatergic and GABAergic inputs develop concurrently, with only a slight lead held by covert NMDAR-mediated inputs (as revealed in Mg^2+^-free saline). Calcium responses mediated by NMDARs and GABA_A_Rs then increase in amplitude over the subsequent 2 days of development (to d9) and then decrease (to d11), whereas those mediated by AMPARs do not change significantly over the same time period. As a result, the relative contributions change, and that of the GABA_A_R-mediated component becomes minor. This time course was the same for responses in both the lateral and medial ipsilateral ROIs, and therefore is likely to be a general feature of oculomotor MNs.

Plausible explanations for the developmental increase in response amplitude include increased presynaptic terminal density, increased transmitter release, increased receptor number and/or density, and increased number and/or density of voltage-gated calcium channels.

A plausible and rather simple explanation for the developmental decline in response amplitude is that easily fatiguable synapses with high probability of neurotransmitter release (Pr) mature into more robust synapses with lower Pr. This specific notion was addressed through paired-pulse stimulation experiments, discussed in the next section.

### Paired pulse facilitation

We show that paired-pulse stimulation gives a response facilitation that increases as the total synaptic response declines. This indicates that changes that might diminish calcium responses through dilution of calcium or CGDA within an ROI, such as somal growth, increased dendritic branching, decreased cell packing, decreased receptor and calcium channel density and increased calcium buffering or extrusion, all of which might occur simply due to growth and maturation of MNs, are unlikely to explain the decline in response amplitude, for the simple reason that such changes also would be expected to counter a developmental increase in response facilitation.

Assuming that an increase in PPF from one stage to another indicates a lower Pr at the later stage, our results would suggest that the decline in response to single stimuli from d9 to d11 could be due to a decrease in Pr from d9 to d11. However, this assumption may not hold (28). It is possible that closer examination of optically recorded presynaptic calcium transients might shed light on whether changes in neurotransmitter release contribute.

Further investigation is therefore needed to determine what causes both the developmental increase and the subsequent developmental decline in response amplitude. In Table 5 we present a list of factors that could potentially contribute to the developmental decline. Despite the uncertainty on this point, the presence of PPF demonstrates substantial synaptic plasticity in the vestibulo-ocular inputs to OM MNs at the developmental stages studied.

**Table 5:** Potential factors that could contribute to the observed developmental decrease in synaptic response.

**Technical**	**Decrease in efficacy of electrical stimulation**
	Decrease in MN health with increasing thickness of preparation
Presynaptic	Decrease in action potential invasion of synaptic terminals
	Decrease in number of synapses
	Decrease in quantal size (transmitter amount per vesicle)
	Decrease in release probability (Pr)
	Increase in transmitter re-uptake kinetics
Postsynaptic	Decrease in receptor number, density or calcium conductance
	Decrease in excitability (affecting activation of VGCC)
	Decrease in VGCC number, density or calcium conductance
	Change in the types of VGCCs expressed
	Decrease in CICR
	Increase in calcium buffering or extrusion

### Summary

The functional development of synaptic inputs from hodologically defined vestibulo-ocular projection neuron groups to extraocular MNs in the oculomotor nuclear complex was assessed by recording postsynaptic calcium responses following stimulation of presynaptic axons in *ex vivo* preparations of the chicken embryo brain stem. Synaptic responses appear shortly after anatomical contact (the structural development of axon terminals and MN dendrites will be described in a separate report). As expected from prior characterization of vestibulo-ocular reflex circuitry, the inputs are both glutamatergic and GABAergic. GABA_A_R-mediated responses are presumed to be depolarizing but are functionally inhibitory in most preparations. Glutamatergic inputs are mediated both by AMPA receptors and NMDA receptors, and NMDAR-mediated responses predominate over AMPAR- and GABA_A_R-mediated responses. Aggregate and receptor-specific responses exhibit dynamic changes in amplitude, rise time and half-width during the developmental period studied. The aggregate response exhibits substantial plasticity in the form of paired-pulse and tetanic facilitation. These results contribute new knowledge about the functional development of the VOR and provide a platform for experimental manipulations to investigate the mechanisms that establish its stereotyped synaptic connectivity.

## Data Availability

The original contributions presented in the study are included in the article, further inquiries can be directed to the corresponding author.
